# A Generalized Correlation for Predicting Ethane, Propane, and Isobutane Hydrates Equilibrium Data in Pure Water and Aqueous Salt Solutions

**DOI:** 10.1002/gch2.201800069

**Published:** 2018-10-26

**Authors:** Azeez G. Aregbe

**Affiliations:** ^1^ Department of Petroleum and Gas Engineering University of Lagos Lagos 101017 Nigeria; ^2^ College of Petroleum Engineering China University of Petroleum (East China) 266580 China

**Keywords:** equilibrium conditions, ethane, high pressure, hydrate, isobutane, propane, salts

## Abstract

Hydrate formation can cause serious problems in hydrocarbon exploration, production, and transportation, especially in deepwater environments. Hydrate‐related problems affects the integrity of the deepwater platforms, leads to equipment blockages, and also increases operational costs. In order to solve these problems, salts are used as thermodynamic inhibitors and also mixed with the drilling fluids in most drilling processes. A comprehensive understanding of hydrate formation in aqueous salt solutions is vital to overcome these problems. Statistical thermodynamic models are commonly used to predict hydrate formation conditions in different aqueous solutions. However, these models involve rigorous computations and are restricted to certain conditions. They give inaccurate predictions of hydrate equilibrium conditions for high‐temperature, high‐pressure, and high‐salinity systems. Therefore, it is paramount to develop a simple‐to‐use and reliable prediction tool. In this work, an empirical correlation is developed and successfully used to predict the equilibrium conditions of ethane, propane, and isobutane hydrates in pure water and aqueous solutions of sodium chloride, potassium chloride, calcium chloride, and magnesium chloride. Experimental data on hydrate formation conditions for these components are regressed and a generalized correlation is obtained. The predictions in this work show excellent agreement with all the experimental data in the literature.

## 1 Introduction

Gas hydrate is a solid crystalline compound that is formed when water molecules and light molecules mostly hydrocarbon compounds exist together at relatively low temperature and high pressure. The water molecules form cage‐like structure that surrounds the light molecules commonly referred to as the guest molecules. The hydrate crystal structure is held together by hydrogen bonding. The presence of these guest molecules makes the hydrate structure slightly stronger than the normal ice structure formed by water molecules. Examples of compounds that form hydrates are carbon dioxide, hydrogen sulfide, methane, ethane, propane, butanes, and nitrogen. It was reported the n‐butane also forms hydrate but it is extremely unstable.^1^ Normal hydrocarbon compounds larger than n‐butane are generally classified as nonhydrate formers. Gas hydrates are divided into sI, sII, and sH structures depending on the type/size of hydrate formers. The formation of hydrate is affected by temperature, pressure, salinity, agitation, and surface area available for crystal growth and agglomeration. Gas hydrates are mostly stable at temperature range 273–300 K and at pressures above 0.6 MPa.^1^, ^2^ Hydrate formation can cause serious problems in hydrocarbon exploration, production, and transportation, most especially in deepwater environments. Hydrate‐related problems will affect the integrity of the deepwater platforms, lead to equipment/pipeline blockages, and also increase operational costs. In order to solve these problems, salts such as sodium chloride are used as thermodynamic inhibitors and also mixed with the drilling fluids in most drilling processes. The formation of hydrate can be prevented by using different techniques. One of these techniques involves the removal of water from the system through dehydration or by using a separator. Another method of preventing the formation of hydrate is to operate outside the hydrate phase envelope (formation region). Hydrate formation can also be prevented by using inhibitors or electrolytes such as salt, methanol, or glycols. A comprehensive knowledge of hydrate formation in pure water and aqueous solutions of these inhibitors is vital to maximize the efficiencies of these preventive techniques.^1^, ^2^, ^3^, ^4^

Accurate prediction of hydrate formation conditions is paramount for the design of any hydrocarbon facility, especially in deepwater and ultra‐deepwater systems. This is because the salinity, temperature, and pressure conditions of these systems are suitable for hydrate formation/dissociation. Experiments have been conducted by different researchers to investigate the formation conditions of hydrates in pure water and the presence of single and mixed inhibitors. Buleiko et al.^1^ measured the formation conditions of n‐butane, i‐butane, and their mixtures in the presence of water‐filled quartz powder. They also measured the heat capacity, enthalpy, and temperature derivative of pressure at isochoric conditions for mixed systems (water and n‐butane, water and i‐butane, water, n‐butane, and i‐butane). They used the measured parameters to design the phase equilibria of hydrates formed by i‐butane and mixtures of n‐butane and i‐butane. They also discovered the quadruple points of i‐butane hydrate and the quintuple point of the hydrate formed by the mixture of n‐butane and i‐butane. It was discovered that n‐butane is not a hydrate former but has a significant effect on the phase equilibrium of hydrate formed by the mixtures of n‐butane and i‐butane. It was also reported that a three‐component system comprising water, n‐butane, and i‐butane with five phases (i.e., ice, liquid water, hydrate, gaseous butanes, and liquid butanes) has zero degree of freedom at the point of quintuple. It was concluded that there is only a single equilibrium point (i.e., temperature, pressure, and composition of butanes) with five phases existing together. Englezos and Bishnoi^3^ investigated the formation conditions of ethane hydrate in the presence of sodium chloride, potassium chloride, calcium chloride, and potassium bromide aqueous solutions. It was discovered that the presence of electrolytes such as salts can shift the equilibrium conditions of hydrates to lower temperatures and higher pressures. And on mass basis, sodium chloride is the strongest among the chloride salts considered in their work. They also compared their experimental results with those available in the literature and good agreement was recorded. Yasuda and Ohmura^4^ studied the phase equilibria of ethane and propane hydrates at temperatures below the ice point. They also investigated the effects of liquid hydrocarbons on hydrate formation conditions at temperatures above the ice point. Three phase equilibrium curves (ice‐liquid water‐vapor) were obtained for the both ethane and propane. They used batch, isochoric method to determine the equilibrium conditions of hydrates. It was reported that the system temperature returned to its original value after hydrate formation/dissociation while the system pressure did not return to its original value. A significant difference between the original and final pressures was recorded when a large quantity of hydrate crystals was formed in the system. It was discovered that there is continuity of equilibrium data below and above the quadruple point, and the formation conditions determined experimentally at the quadruple point coincided with those obtained from theoretical calculations. Mohammadi et al.^5^ conducted experiments to measure the formation conditions of ethane, and propane hydrates in the presence of sodium chloride, potassium chloride, and calcium chloride. They used an isochoric pressure‐search method to determine the equilibrium data of hydrates. It was reported that salts inhibit hydrate formation as a result of the strong electrostatic forces they created when dissolved in water. The dissolution of salts in water will create ions that can distort the hydrogen bonds in the hydrate crystals and inhibit hydrate formation. The degree of temperature suppression is dependent on the type/amount of salts present in solution. Long et al.^6^ studied the inhibiting effects of magnesium chloride on the equilibrium data of ethane hydrate. Constant volume pressure‐search method was used to determine the formation conditions of ethane hydrate in aqueous solution of magnesium chloride. They also measured ethane hydrate formation conditions in pure water to verify the reliability of their experimental apparatus and procedure. Their experimental results followed similar trends with those available in the literature. It was discovered that increase in the concentration of magnesium chloride shifted ethane hydrate equilibrium data to lower temperatures and higher pressures. They also reported that the chloride ions formed when salt dissolves in water have the strongest effects on hydrate formation conditions compared with any of the cations (i.e., Na^+^).

Several thermodynamic models have been developed to predict the formation conditions of hydrates formed in different solutions. These models can predict the equilibrium conditions of hydrates in the presence of single and mixed inhibitors at low‐to‐moderate temperature, pressure, and concentration of inhibitors. The first thermodynamic equation was developed by Van der Waals and Platteeuw.^7^ Their statistical thermodynamic equation is the basic equation used to develop other models. The thermodynamic model was developed by using the basic equation for gas adsorption process. They assumed that the volatile component in the hydrate structure moves in a spherical cavity and the cavity contains only a gaseous molecule. The size of the gaseous molecule is very small compared to that of the hydrate structure and the molecules in all the cavities are independent of one another. The interactions between water molecules and the gaseous components were described with the London dispersion forces, which are relatively weak van der Waal's forces. They combined Lennard‐Jones parameters with the thermodynamic equation to estimate the equilibrium data of hydrates formed by pure gases. Parrish and Prausnitz^8^ modified the thermodynamic equation proposed by van der Waals and Platteeuw^7^ by including the Kihara parameters to account for sphericity of the hydrate structure. Their predictions were compared with the experimental data available in the literature and satisfactory agreement was recorded. John et al.^9^ proposed a modification to van der Waal and Platteeuw model^7^ by including a simple function to account for spherical asymmetric. The deviation of Langmuir constants from ideal conditions was also determined. They calculated the values of the Kihara potentials from the experimental data and their correlation. Excellent agreement was recorded between the result from experimental data and that from the virial coefficient data. Their model is a useful tool for estimating hydrate phase equilibria of any gas mixture except hydrogen‐rich systems. Chen and Guo^10^ presented a different approach to the prediction of hydrate equilibrium conditions. A two‐step process that involves the formation of hydrate by a quasi‐chemical reaction and encaging of volatile components by adsorption process was proposed. They used the proposed mechanism to establish a simple prediction method for estimating the formation conditions of hydrates formed by pure gases and gas mixtures. Their predictions showed satisfactory agreement with most of the experimental data available. The statistical thermodynamic approach is commonly used for the prediction of hydrate equilibrium data because there are limited alternatives. These models are not easy‐to‐use, complex, and involve rigorous computations. They are also not effective in predicting hydrate formation conditions of high‐temperature, high‐pressure, and high‐salinity systems.^11^ Their prediction errors increase with increase in temperature, pressure, and salinity. In addition, the commercial hydrate prediction programs that are widely used in the industry and academic are developed by using the statistical thermodynamic approach. The prediction errors of the thermodynamic models are reflected in the predictions of these programs especially for high‐temperature, high‐pressure, and high‐salinity systems.^11^

In addition to the thermodynamic models, there are several empirical correlations used to predict the degree of equilibrium temperature reduction of hydrates caused by the presence of inhibitors. Hammerschmidt^12^ developed the basic correlation for predicting hydrate suppression temperature in the presence of common inhibitors such as methanol and glycol. The basic correlation was developed from the principle of freezing point depression. The empirical correlation is shown in Equation (1) and is applicable to ≤30 wt% methanol or ethylene glycol and ≤20 wt% of other glycols. In the equation, Δ*T* (°C) is hydrate temperature suppression, *M* (g mol^−1^) is the molar mass of the inhibitor, *W* (wt%) is the weight percent of the inhibitor, and *K*_H_ = 1297

(1) $$\Delta T=\frac{K_{H}W}{M\left(100-W\right)}$$

Mohammadi and Tohidi^13^ proposed an empirical correlation for predicting the suppression temperature of hydrate equilibrium data caused by the presence of salts and other inhibitors. The proposed correlation is shown in Equation (2) and includes the amount of inhibitor and/or salts present in the system. The parameter *x*_solute_ is the inhibitor mole fraction, *W_i_* is the salt weight percent, and *a*, *b*, *c*_1_, *c*_2_, and *c*_3_ are constants of the equation

(2) $$\Delta T=-a\left[\ln\left(1-x_{\mathrm{solute}}\right)+bx_{\mathrm{solute}}^{2}+\sum \left(c_{1,i}W_{i}+c_{2,i}W_{i}{}^{2}+c_{3,i}W_{i}{}^{3}\right)\right]$$

Bahadori et al.^14^ developed an empirical correlation for estimating hydrate equilibrium pressures of alkanes in the presence of sodium chloride, ethylene glycol, methanol, and triethylene glycol. The empirical correlation was developed by using some of the experimental data reported in the literature. The proposed correlation is shown in Equation (3). In the correlation, *P* (MPa) is the equilibrium pressure of hydrate, *T* (K) is the equilibrium temperature of hydrate, and *a*, *b*, *c*, and *d* are the coefficients of the correlation. The values of coefficients depend on the amount of inhibitor present in the systems and are determined by tuning several parameters. The tuned parameters contain ≈15‐digit numbers and are prone to rounding off errors

(3) $$P=a+bT+cT^{2}+dT^{3}$$

Aregbe^11^ proposed a generalized correlation for calculating the formation conditions of methane hydrate in pure water and aqueous solutions of sodium chloride, potassium chloride, calcium chloride, and magnesium chloride. The generalized correlation was developed by regressing all the experimental data available in the literature for methane hydrate formation in the presence of different solutions. The generalized correlation is shown in Equation (4). In the equation, *P*_eq_ (MPa) is the formation pressure of hydrate, *T*_eq_ (K) is hydrate formation temperature, *A_x_*, *B_x_*, *C_x_*, *D_x_*, *E_x_*, and *F_x_* are coefficients of the equations. The values of these coefficients depend on the type/quantity of salts dissolved in water. The generalized correlation can accurately predict the equilibrium data of methane hydrate in low, moderate, and high temperature, pressure, and salinity systems

(4) $$P_{\mathrm{eq}}=A_{x}\left[T_{\mathrm{eq}}^{5}\right]+B_{x}\left[T_{\mathrm{eq}}^{4}\right]+C_{x}\left[T_{\mathrm{eq}}^{3}\right]+D_{x}\left[T_{\mathrm{eq}}^{2}\right]+E_{x}\left[T_{\mathrm{eq}}\right]+F_{x}$$

In this work, the empirical correlation in Equation (4) is further simplified and extended by using the available experimental data in the literature for ethane, propane, and isobutane hydrates. A simplified and easy‐to‐use correlation is developed for accurate prediction of the equilibrium data of ethane, propane, and i‐butane hydrates in pure water and aqueous solutions of sodium chloride, potassium chloride, calcium chloride, and magnesium chloride at pressure up to 500 MPa.

## 2 Generalized Correlation

Most statistical thermodynamic models are combined with the cubic equation of state (Eos) to obtain accurate prediction of hydrate equilibrium data. But these Eos are not reliable for systems containing water, electrolytes, methanol, or glycol due to their strong specific interactions.^14^ Different assumptions are also made in developing these models and they often involve rigorous computations. These models also give inaccurate predictions for high temperature, pressure, and salinity systems.^11^ Hence, a simple, easy‐to‐use, and reliable prediction method is needed for accurate estimating of hydrate equilibrium data for any type of systems. In this work, a generalized correlation is developed by regression all the available experimental data^15^, ^16^, ^17^, ^18^, ^19^, ^20^, ^21^, ^22^, ^23^, ^24^, ^25^, ^26^, ^27^, ^28^, ^29^, ^30^, ^31^, ^32^, ^33^, ^34^, ^35^, ^36^ in the literature. The generalized correlation is shown in Equation (5). The generalized correlation can be used to predict the formation conditions of ethane, propane, and i‐butane hydrates in pure water and aqueous solutions of chloride salts. Ethane hydrate formation in pure water and 0%–20 wt% single sodium chloride, potassium chloride, calcium chloride, and magnesium chloride aqueous solutions at temperature range of −72.35–52.16 °C and pressure range of 0.01–500.39 MPa are considered. Also, propane hydrate formation in pure water and 0%–20 wt% single sodium chloride, potassium chloride, and calcium chloride aqueous solutions at temperature range of −25.25–5.75 °C and pressure range of 0.05–0.58 MPa are considered. In addition, isobutane hydrate formation in pure water and 0%–20 wt% sodium chloride aqueous solutions at temperature range of −38.39–1.97 °C and pressure range of 0.01–0.17 MPa are considered in this work. The temperature range, pressure range, and type of salts considered are based on the experimental data reported in the literature

(5) $$P_{\mathrm{eq}}=A_{x}T_{\mathrm{eq}}^{3}+B_{x}T_{\mathrm{eq}}^{2}+C_{x}T_{\mathrm{eq}}+D_{x}$$

In the equation above, *P*_eq_ is hydrate formation pressure (MPa) and *T*_eq_ is the formation temperature of hydrate. The variables *A_x_*, *B_x_*, *C*_*x*,_ and *D_x_* depend on the type/concentration of salt present in the system. The value of *A_x_* can be determined by using Equation (6)

(6) $$A_{x}=A_{4}x^{4}+A_{3}x^{3}+A_{2}x^{2}+A_{1}x+A_{0}$$

The variable “*x*” is the weight percent of salt in aqueous phase. Similarly, the values of *B_x_*, *C_x_*, and *D_x_* can be calculated by using Equations (7)–(9)

(7) $$B_{x}=B_{4}x^{4}+B_{3}x^{3}+B_{2}x^{2}+B_{1}x+B_{0}$$

(8) $$C_{x}=C_{4}x^{4}+C_{3}x^{3}+C_{2}x^{2}+C_{1}x+C_{0}$$

(9) $$D_{x}=D_{4}x^{4}+D_{3}x^{3}+D_{2}x^{2}+D_{1}x+D_{0}$$

The tuned parameters are *A*_0_ − *A*_4_, *B*_0_ − *B*_4_, *C*_0_ − *C*_4_, and *D*_0_ − *D*_4_ and their values depend on the concentration of salt in the aqueous phase. In pure water systems, the value of “*x*,” weight percent of salt in the aqueous phase is zero. Therefore, the tuned parameters are transformed into constants (*A*, *B*, *C*, and *D*) for hydrate formation in pure water. The values of these tuned constants for each hydrate system are provided in **Table** **1** while the values of the tuned parameters are provided in **Tables** **2**–**5**.

**Table 1:** The tuned constants for hydrate formation in pure water

Component	Phase	*T* range [°C]	*A*	*B*	*C*	*D*
Ethane	*I* − *H* − *V*	−72.35 to −1.25	1.57E‐06	2.96E‐04	0.01967	0.4772
	*L*_w_ − *H* − *V*	0.25 to 15.05	5.91E‐04	2.86E‐04	0.0567	0.5315
	$L_{w}-H-L_{C_{2}H_{6}}$	14.55 to 52.16	−9.44E‐04	0.2802	−1.9652	−25.468
Propane	*I* − *H* − *V*	−28.15 to −0.25	−5.31E‐07	9.75E‐05	7.75E‐03	0.1735
	*L*_w_ − *H* − *V*	0.05 to 5.75	−1.38E‐05	8.35E‐03	0.0252	0.1657
Isobutane	*I* − *H* − *V*	−38.39 to −0.02	1.31E‐06	1.405E‐04	6.16E‐03	0.1121
	*L*_w_ − *H* − *V*	0.05 to 1.97	0.0022	−0.0025	0.026	0.112

**Table 2:** The tuned parameters (*A_n_*) for hydrate formation in aqueous salt solution

Component	Salt type	*A*_0_	*A*_1_	*A*_2_	*A*_3_	*A*_4_
Ethane	NaCl	−3.756E‐03	1.4021E‐03	−1.118E‐04	3.476E‐06	0.00
	KCl	−0.008398	0.00282	−2.6043E‐04	6.8973E‐06	0.00
	CaCl_2_	0.0001915	0.0001614	−1.5164E‐05	4.336E‐07	0.00
	MgCl_2_	−0.005422	0.012526	−5.2187E‐03	6.6731E‐04	−2.5989E‐05
Propane	NaCl	−0.0101445	6.278E‐03	−1.1041E‐03	7.6546E‐05	−1.7324E‐06
	KCl	−0.04113	0.012898	−1.2538E‐03	3.4334E‐05	0.00
	CaCl_2_	0.01473	−0.005807	8.385E‐04	−3.385E‐05	0.00
Isobutane	NaCl	−0.10032	0.17166	−0.062622	8.0665E‐03	−3.3832E‐04

**Table 3:** The tuned parameters (*B_n_*) for hydrate formation in aqueous salt solution

Component	Salt type	*B*_0_	*B*_1_	*B*_2_	*B*_3_	*B*_4_
Ethane	NaCl	−0.09077	0.03507	−4.0705E‐03	1.3988E‐04	0.00
	KCl	0.0496	−0.01401	0.00129	−3.445E‐05	0.00
	CaCl_2_	0.02699	−0.008735	8.328E‐04	−2.107E‐05	0.00
	MgCl_2_	0.159397	−0.33375	0.14062	−0.018304	7.3378E‐04
Propane	NaCl	−0.005992	0.012285	−3.242E‐03	3.084E‐04	−6.8379E‐06
	KCl	−0.20602	0.07934	−7.995E‐03	2.0644E‐04	0.00
	CaCl_2_	0.22505	−0.08943	0.01097	−3.926E‐04	0.00
Isobutane	NaCl	−0.02448	0.08077	−3.309E‐02	6.3344E‐03	−4.433E‐04

**Table 4:** The tuned parameters (*C_n_*) for hydrate formation in aqueous salt solution

Component	Salt type	*C*_0_	*C*_1_	*C*_2_	*C*_3_	*C*_4_
Ethane	NaCl	−0.13775	0.07335	−0.00756	2.8364E‐04	0.00
	KCl	0.18305	−0.04312	0.004568	−1.244E‐04	0.00
	CaCl_2_	0.2012	−0.04708	0.004598	−1.101E‐04	0.00
	MgCl_2_	−1.29658	2.83762	−1.20184	0.158156	−0.0064314
Propane	NaCl	0.038158	0.009744	−0.002953	1.3082E‐04	2.284E‐05
	KCl	−0.38779	0.13856	−0.01033	1.174E‐04	0.00
	CaCl_2_	0.2703	−0.10133	0.01345	−4.685E‐04	0.00
Isobutane	NaCl	0.26269	−0.39536	0.227147	−0.049594	0.0034046

**Table 5:** The tuned parameters (*D_n_*) for hydrate formation in aqueous salt solution

Component	Salt type	*D*_0_	*D*_1_	*D*_2_	*D*_3_	*D*_4_
Ethane	NaCl	0.03232	0.1908	−0.01852	7.268E‐04	0.00
	KCl	0.4498	0.01832	0.001085	−3.025E‐05	0.00
	CaCl_2_	1.6317	−0.3812	0.0376	−9.342E‐04	0.00
	MgCl_2_	4.2652	−7.8568	3.36195	−0.44623	0.018343
Propane	NaCl	0.6519	−0.34329	0.0912	−0.009625	3.875E‐04
	KCl	0.3714	−0.1035	0.01892	−8.295E‐04	0.00
	CaCl_2_	−0.0915	0.09668	−0.008396	4.329E‐04	0.00
Isobutane	NaCl	1.244	−1.7687	0.83927	−0.15164	0.0090718

## 3 Results and Discussion

**Figure 1:**
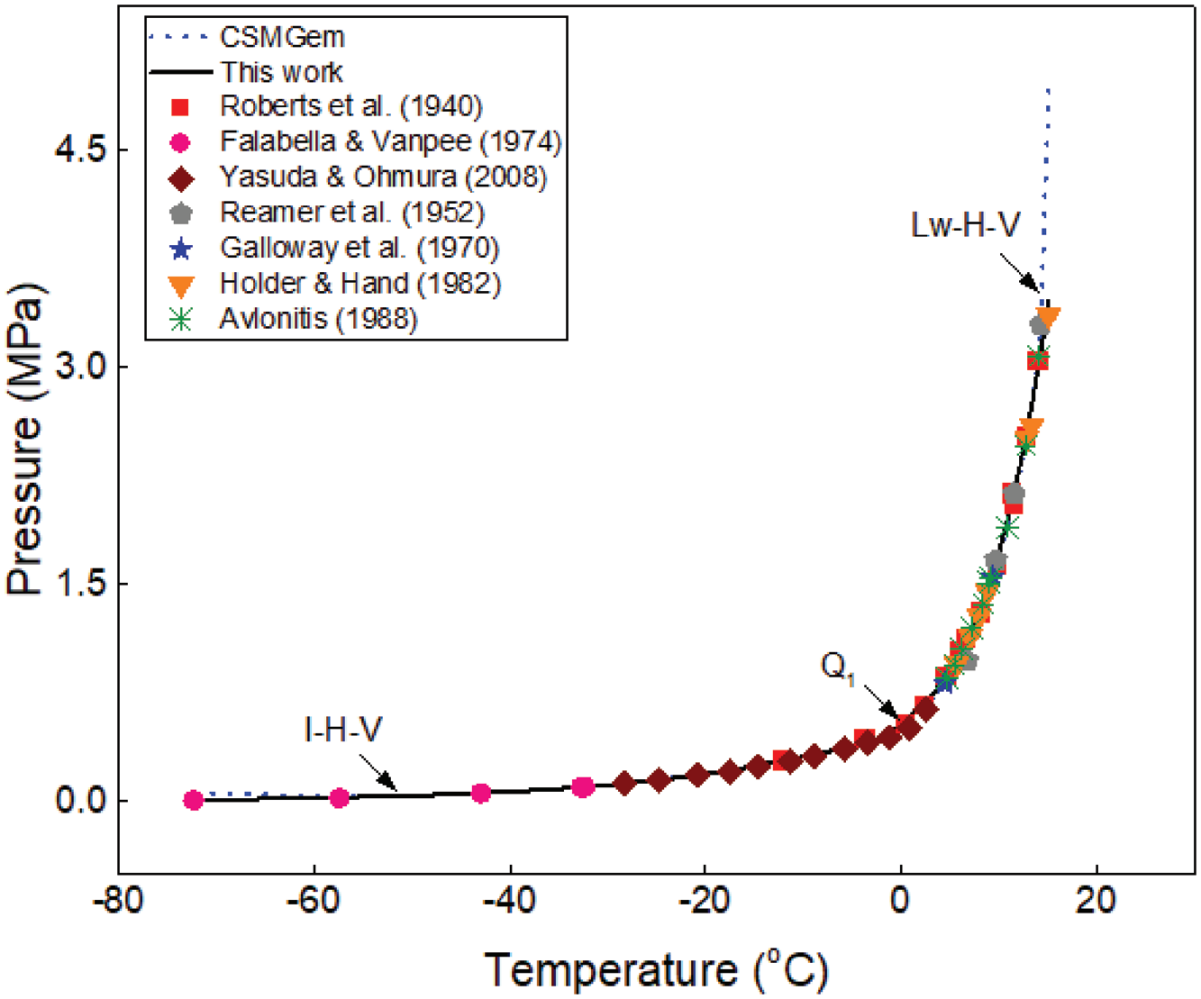
Plot of ethane hydrate equilibrium data in pure water systems. Comparison of experimental data, predictions in this work, and data from CSMGem.

**Figure 2:**
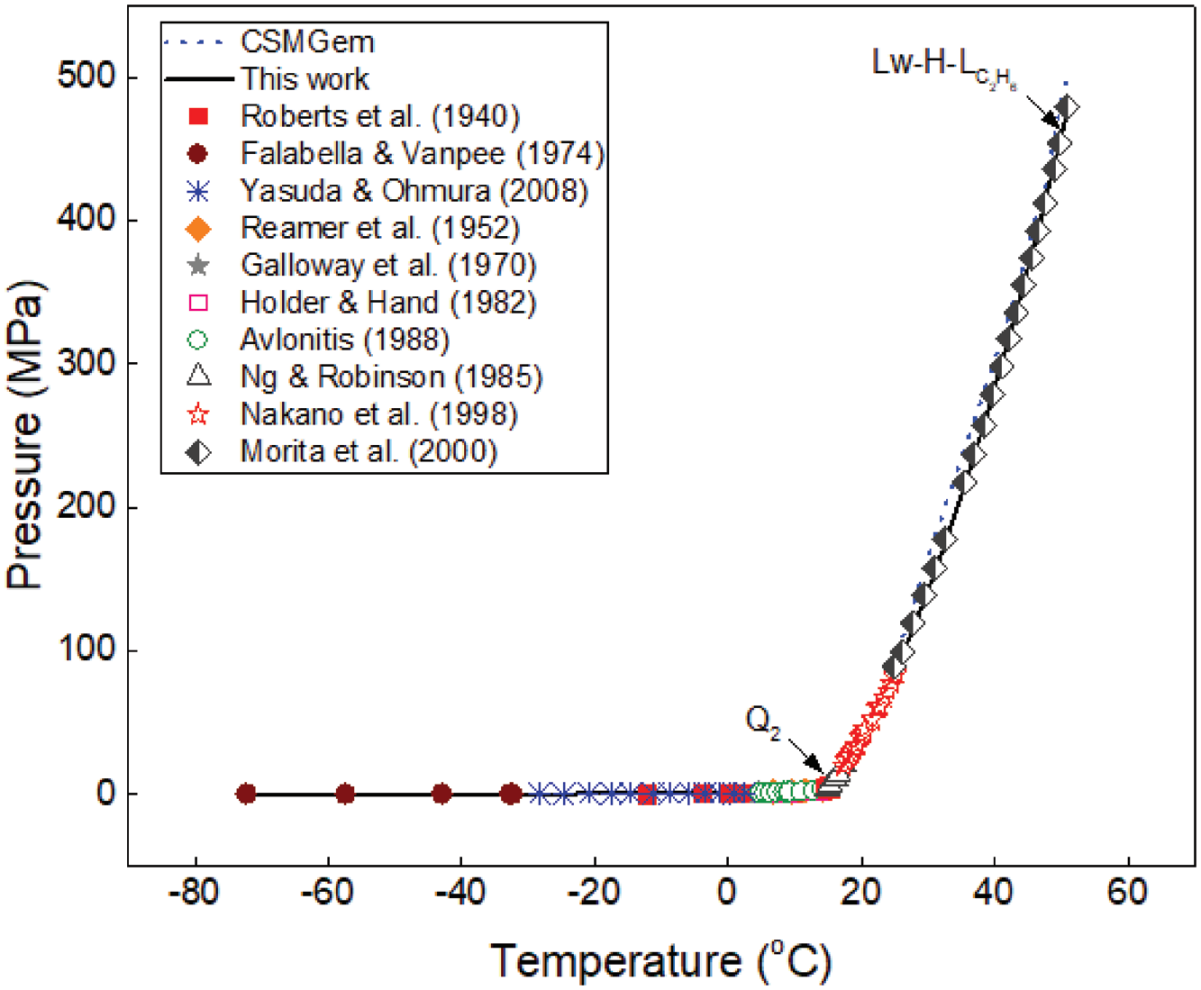
Plot of ethane hydrate equilibrium data in pure water systems. Comparison of experimental data, predictions in this work, and data from CSMGem.

**Figure 3:**
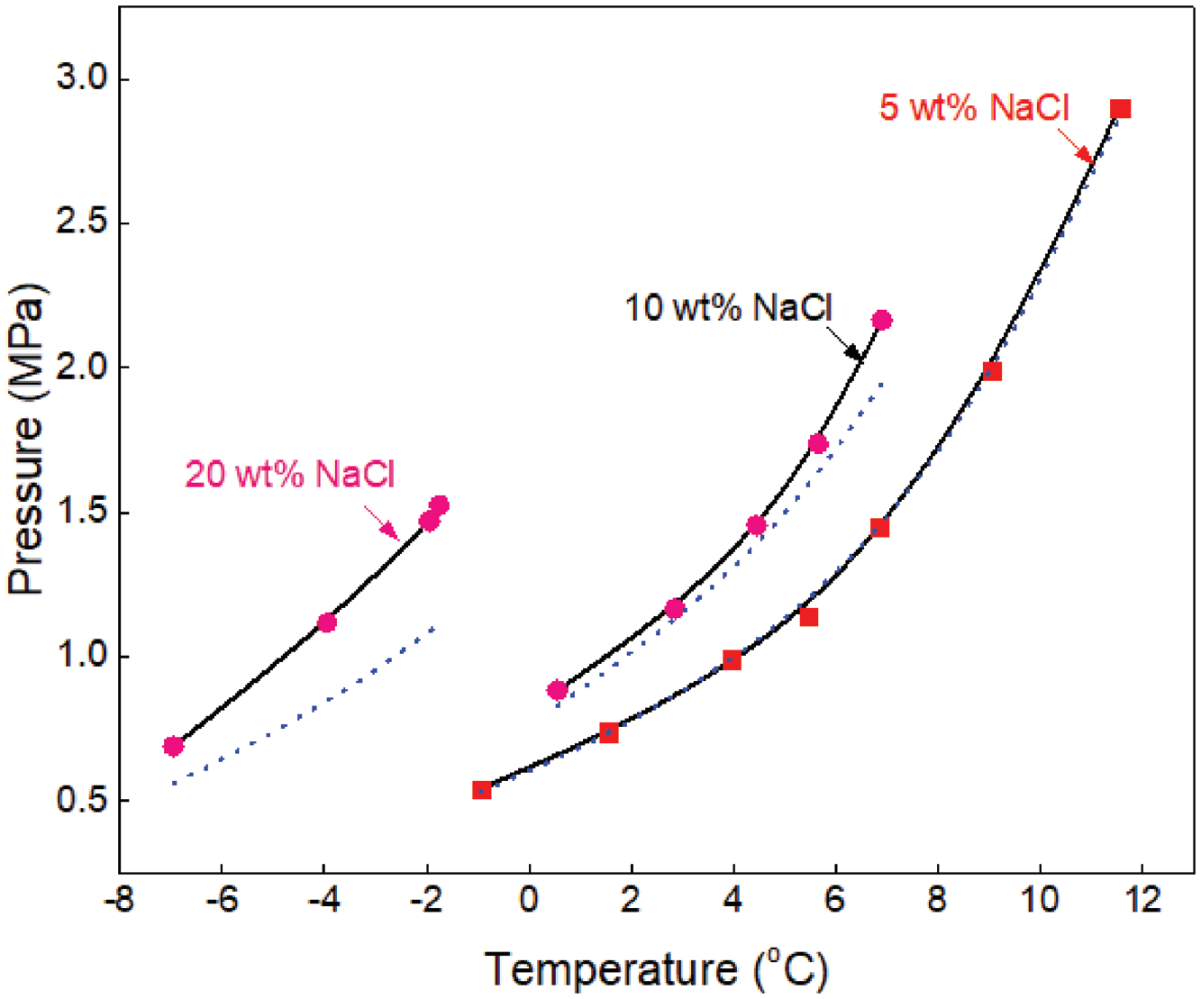
Ethane hydrate equilibrium data in sodium chloride aqueous solutions. Experimental data: rectangle‐red^5^ and circle‐pink.^23^ Prediction: dot‐blue^36^ and line‐black (this work).

**Figure 4:**
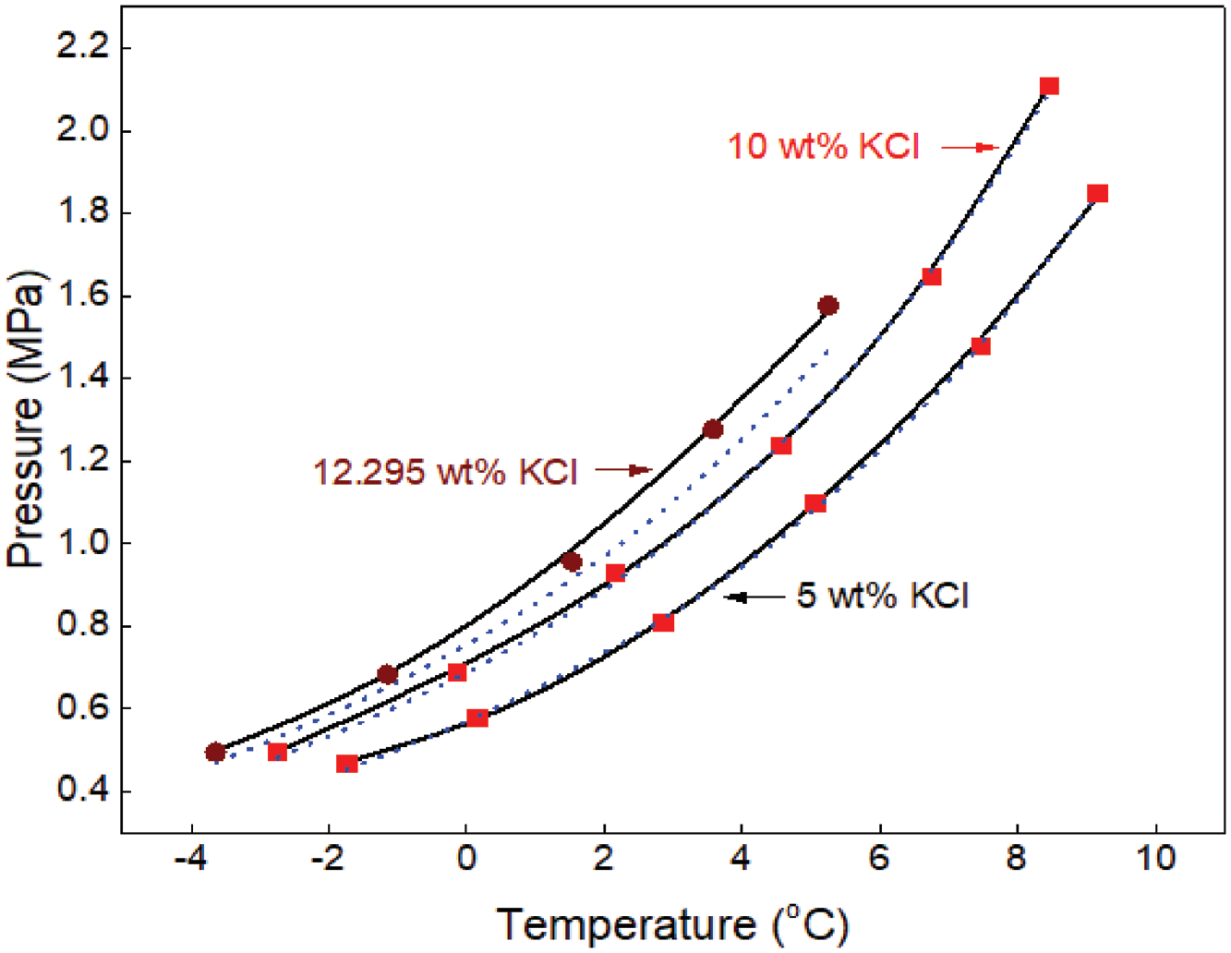
Ethane hydrate equilibrium data in potassium chloride aqueous solutions. Experimental data: rectangle‐red^5^ and circle‐brown.^3^ Prediction: dot‐blue^36^ and line‐black (this work).

**Figure 5:**
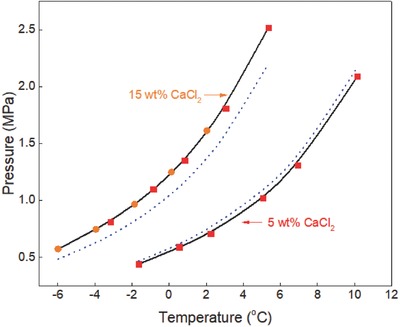
Ethane hydrate equilibrium data in calcium chloride aqueous solutions. Experimental data: rectangle‐red^5^ and circle‐orange.^3^ Prediction: dot‐blue^36^ and line‐black (this work).

**Figure 6:**
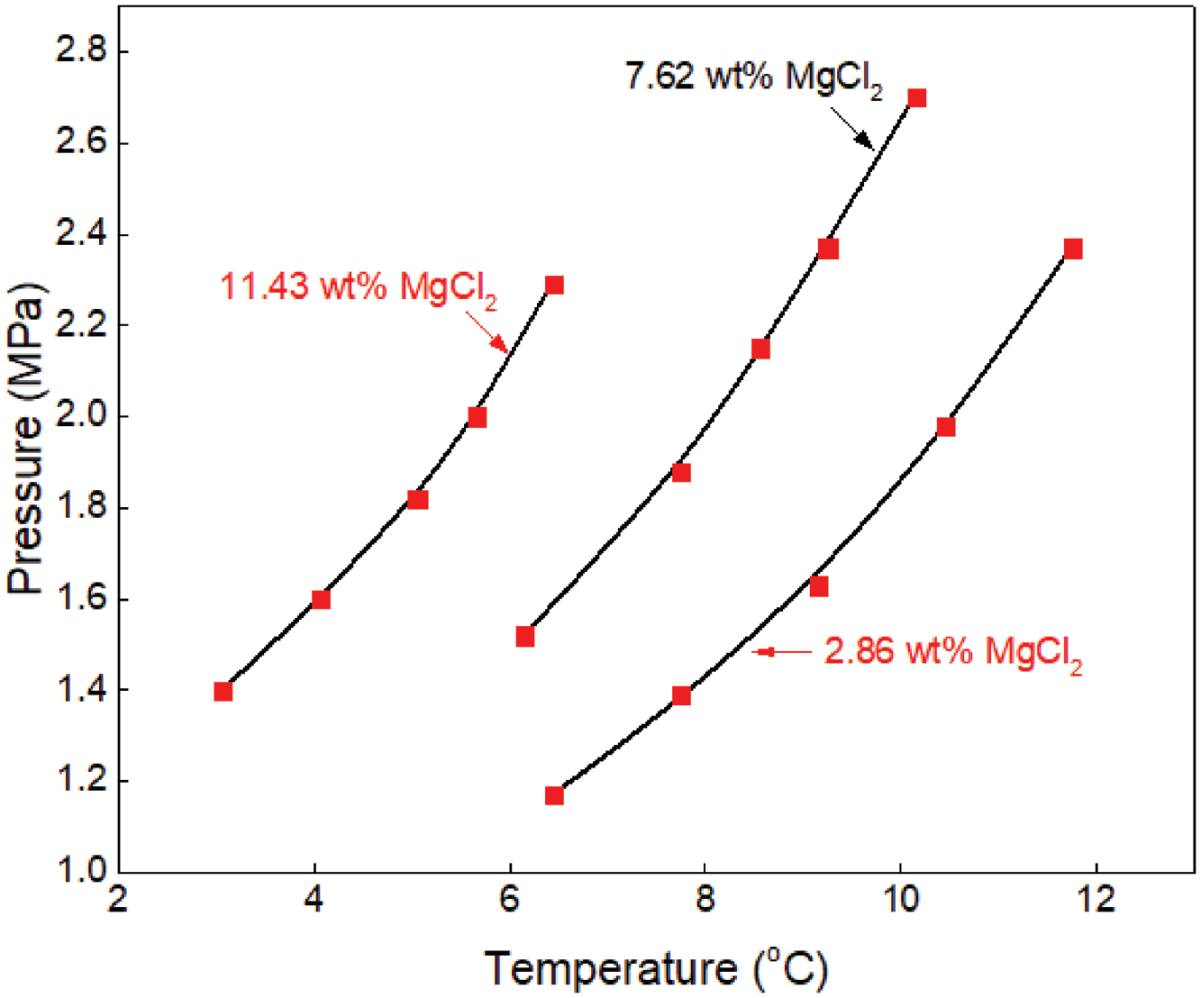
Ethane hydrate equilibrium data in magnesium chloride aqueous solutions. Experimental data: rectangle‐red.^6^ Prediction: line‐black (this work).

**Figure 7:**
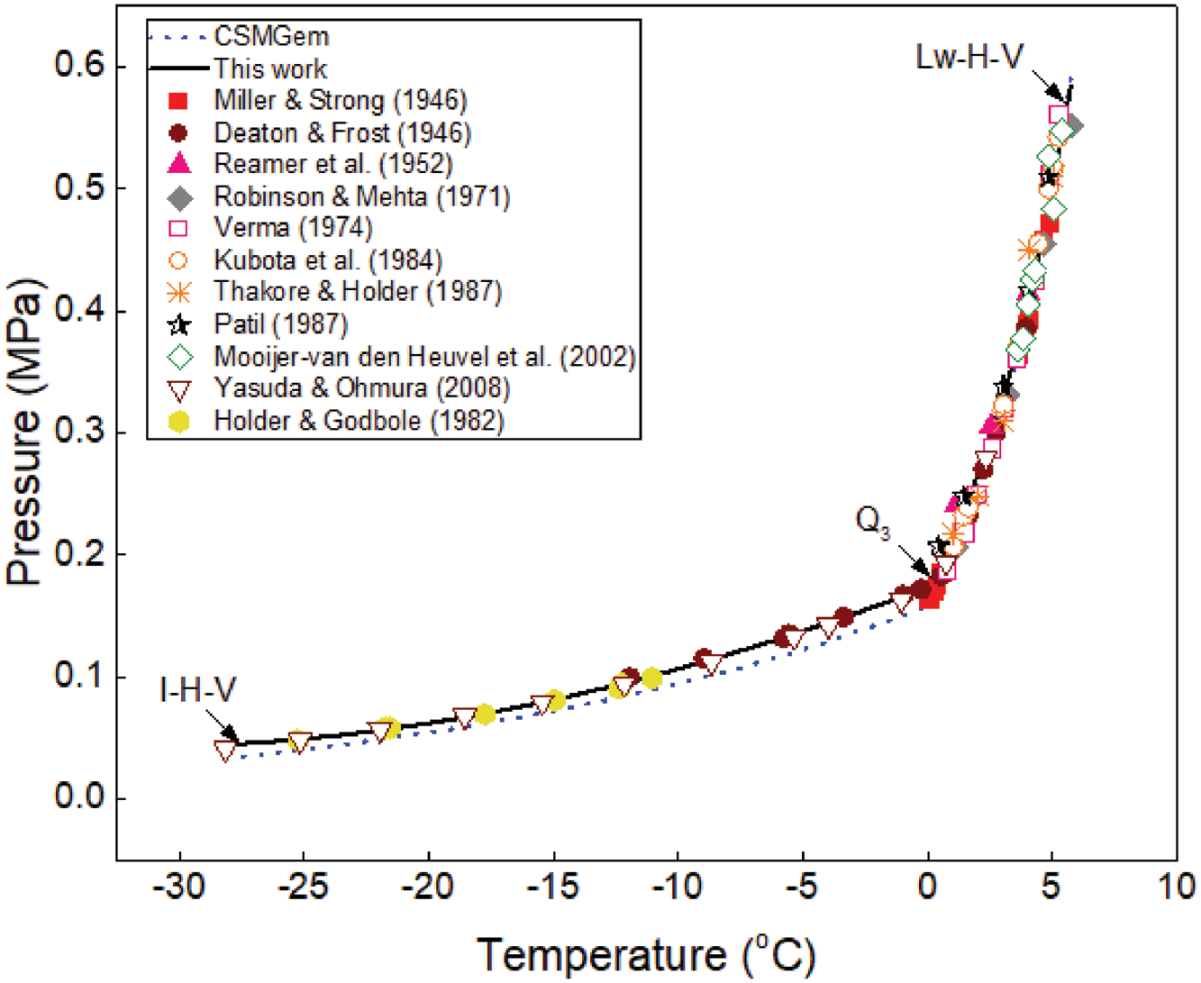
Plot of propane hydrate equilibrium data in pure water systems. Comparison of experimental data, predictions in this work, and data from CSMGem.

**Figure 8:**
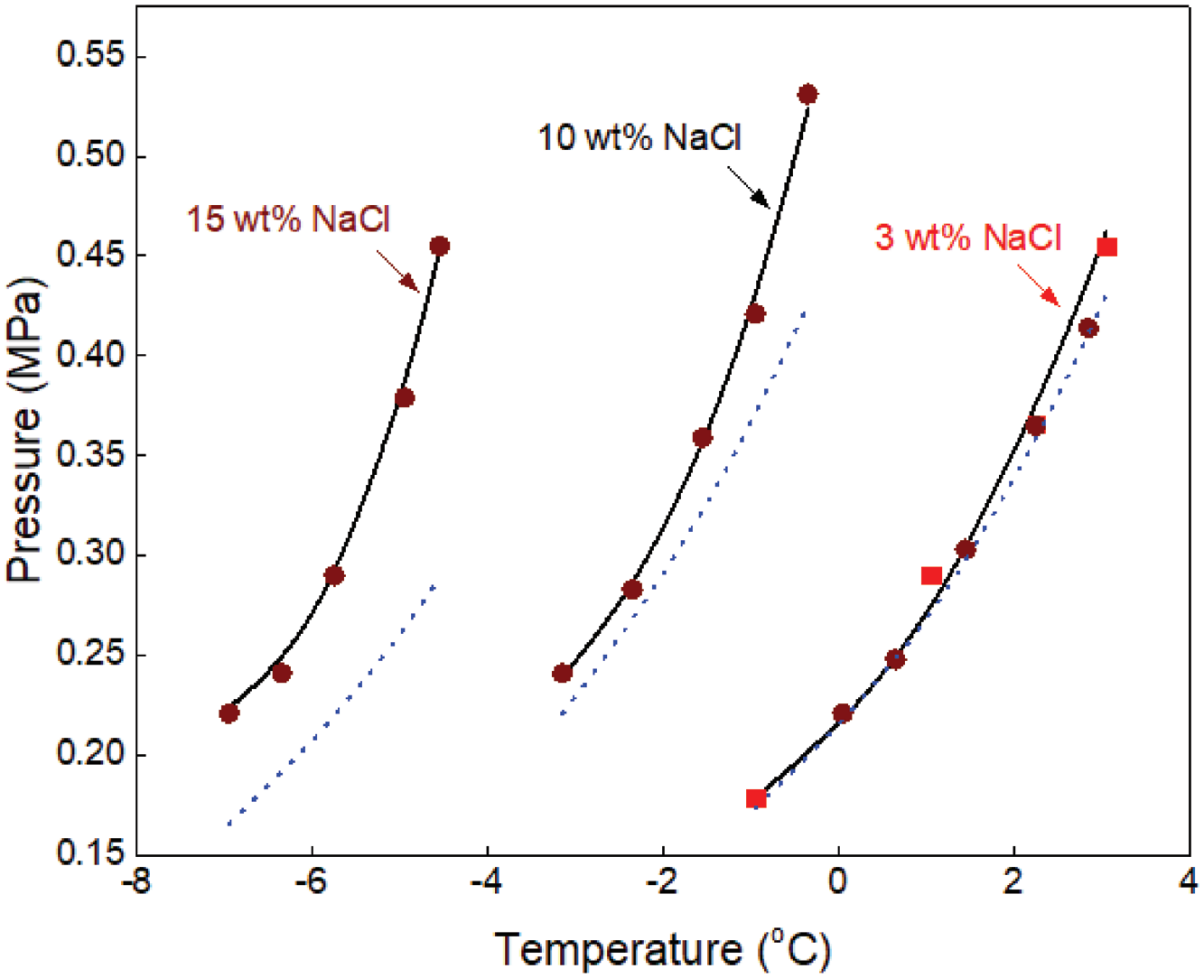
Propane hydrate equilibrium data in sodium chloride aqueous solutions. Experimental data: rectangle‐red^30^ and circle‐brown.^23^ Prediction: dot‐blue^36^ and line‐black (this work).

**Figure 9:**
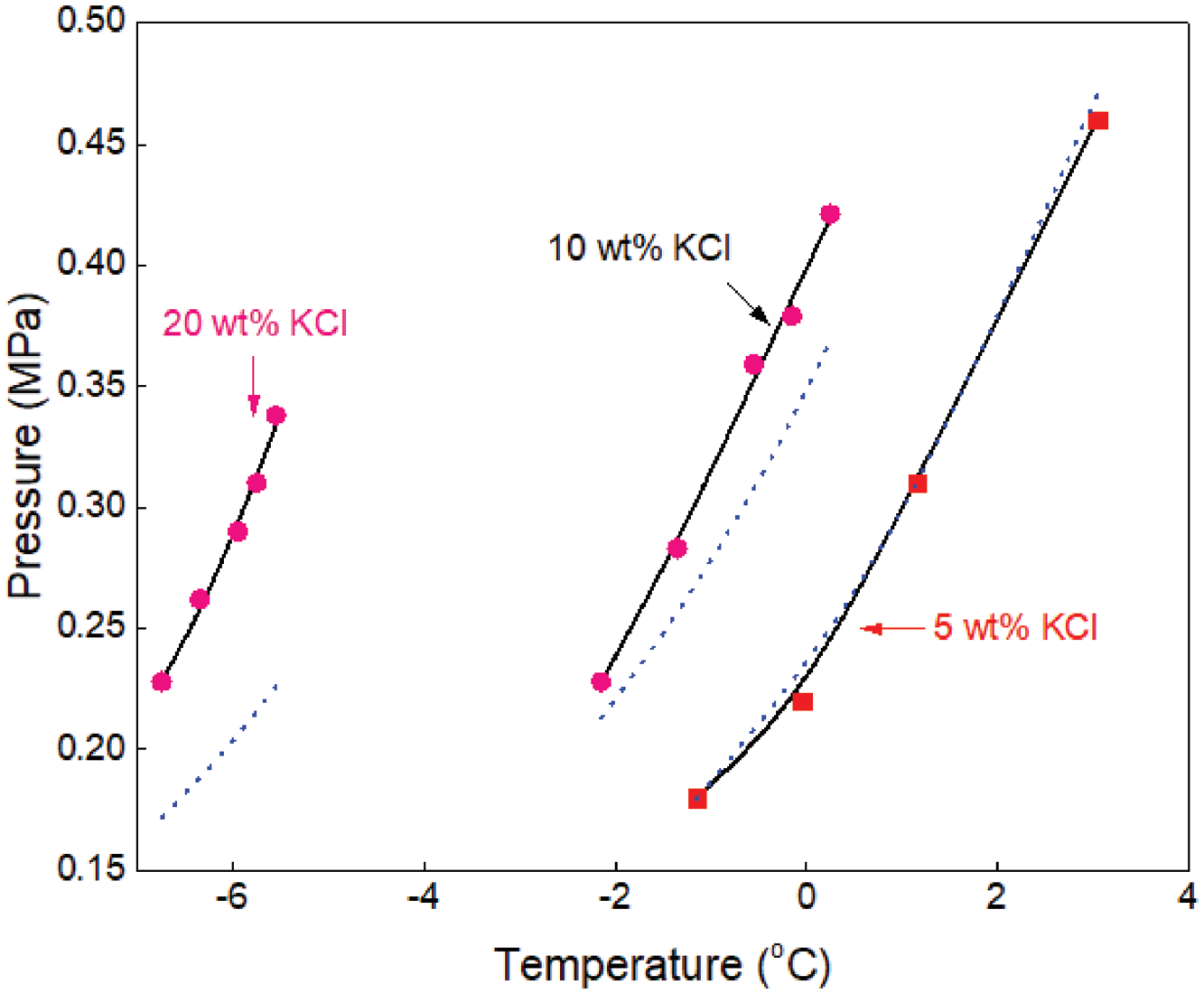
Propane hydrate equilibrium data in potassium chloride aqueous solutions. Experimental data: rectangle‐red^5^ and circle‐pink.^23^ Prediction: dot‐blue^36^ and line‐black (this work).

**Figure 10:**
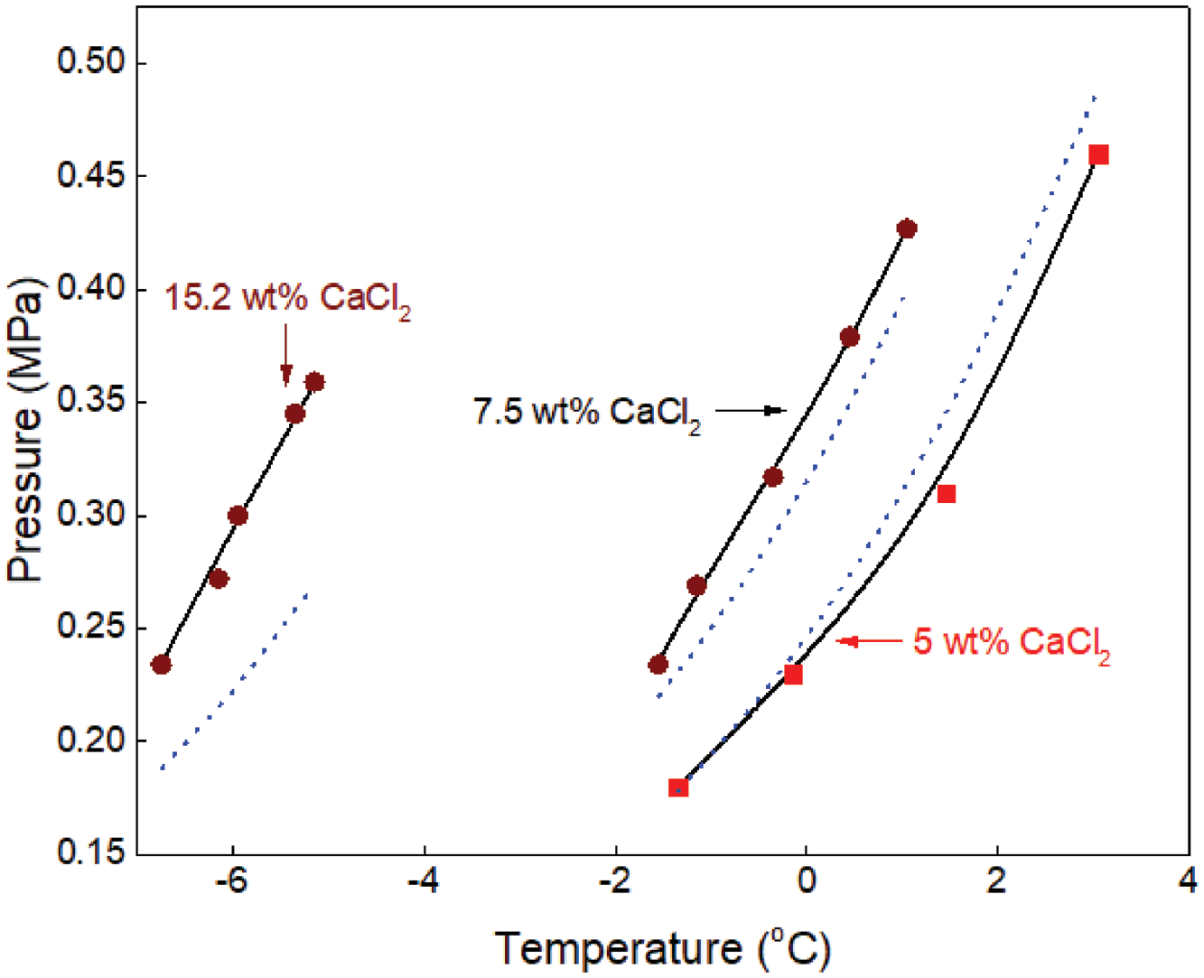
Propane hydrate equilibrium data in calcium chloride aqueous solutions. Experimental data: rectangle‐red^5^ and circle‐brown.^23^ Prediction: dot‐blue^36^ and line‐black (this work).

**Figure 11:**
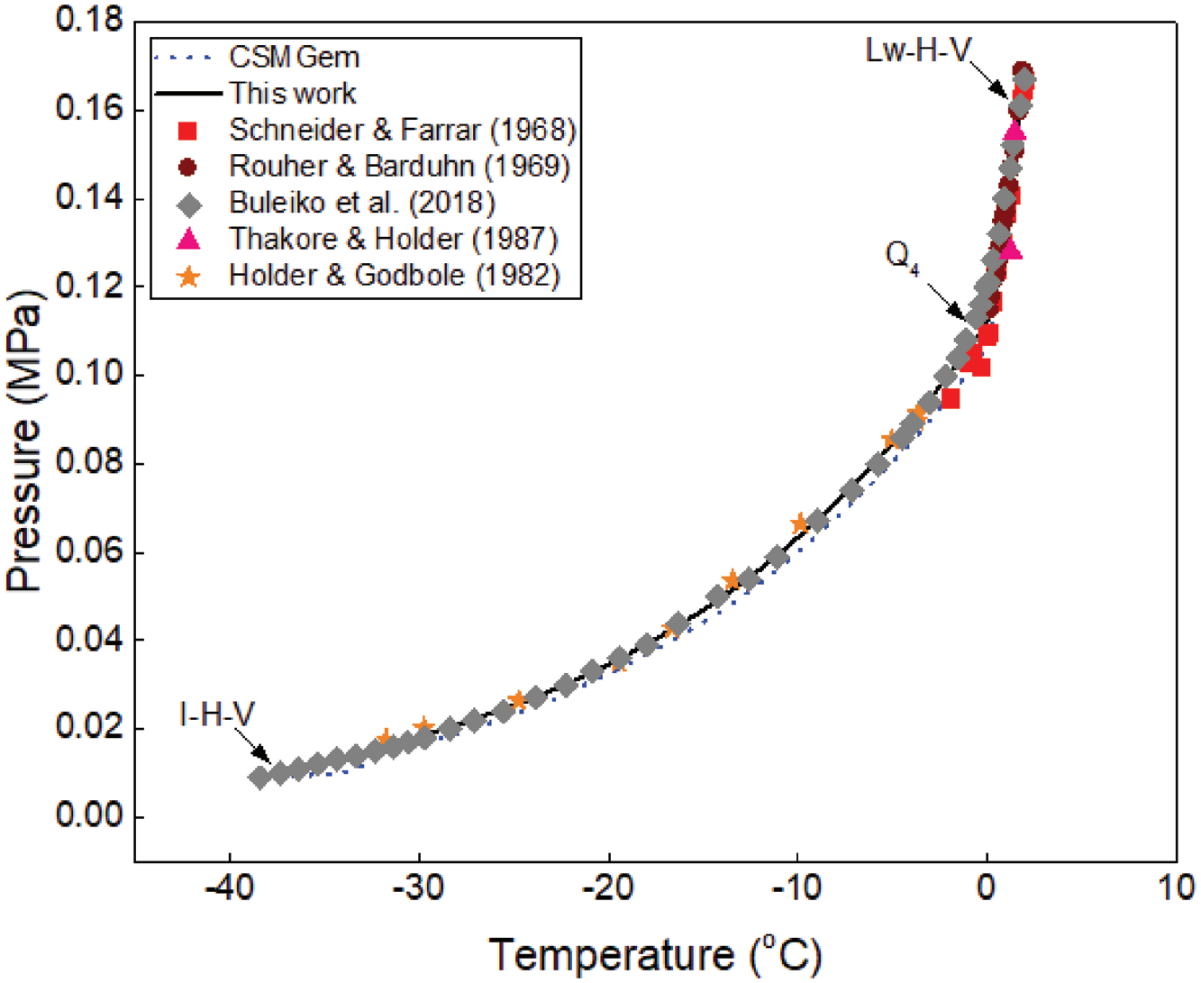
Plot of isobutane hydrate equilibrium data in pure water systems. Comparison of experimental data, predictions in this work, and data from CSMGem.

**Figure 12:**
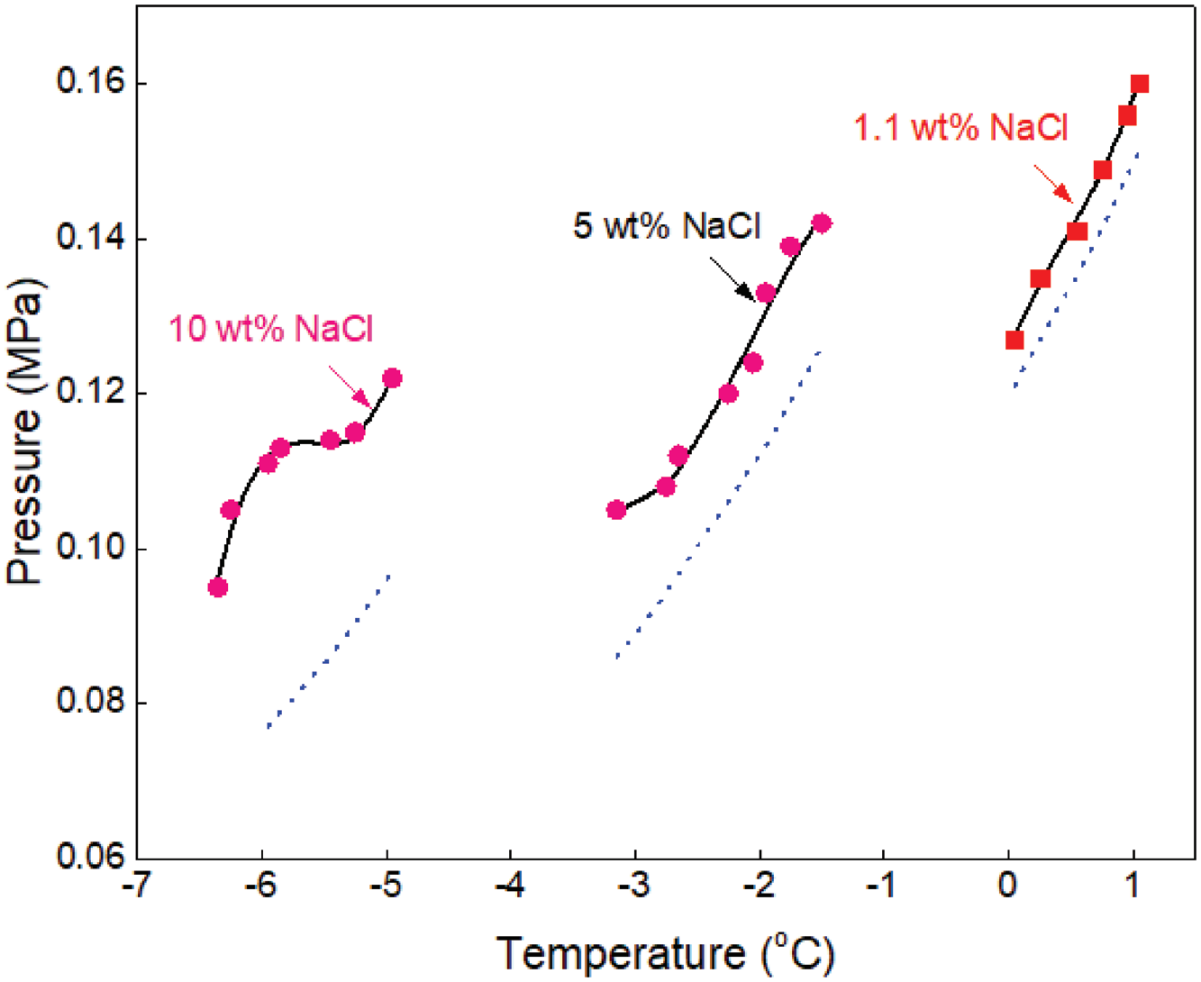
Isobutane hydrate equilibrium data in sodium chloride aqueous solutions. Experimental data: rectangle‐red^33^ and circle‐brown.^35^ Prediction: dot‐blue^36^ and line‐black (this work).

The generalized correlation developed in this work was validated by using different sets of experimental data^15^, ^16^, ^17^, ^18^, ^19^, ^20^, ^21^, ^22^, ^23^, ^24^, ^25^, ^26^, ^27^, ^28^, ^29^, ^30^, ^31^, ^32^, ^33^, ^34^, ^35^, ^36^ available in the literature to ensure the reliability of the correlation. Ethane hydrate formation conditions in pure water and aqueous solutions of sodium chloride, potassium chloride, calcium chloride, and magnesium chloride are shown in **Figures** **1**–**6**. The equilibrium data of propane hydrate in pure water and aqueous solutions of sodium chloride, potassium chloride, and calcium chloride are shown in **Figures** **7**–**10** while the equilibrium conditions of isobutane hydrate in pure water and sodium chloride aqueous solutions are shown in **Figures** **11** and **12**. The trends in these figures show that the equilibrium pressure of hydrate is directly proportional to its equilibrium temperature and the formation conditions of hydrate are strongly dependent on the type/concentration of salts in the system. Hydrate equilibrium data in 15 wt% sodium chloride aqueous solution are not the same as those in 15 wt% magnesium chloride or calcium chloride aqueous solutions. Also, hydrate formation conditions in 3 wt% potassium chloride aqueous solution are different from those recorded in 5 wt% potassium chloride aqueous solution. Thus, in the presence of different type/concentration of salts, the formation conditions of hydrate for any system are unique.

Ethane hydrate equilibrium data in pure water systems are shown in Figures 1 and 2. Experimental data^2^, ^4^, ^15^, ^16^, ^17^, ^18^, ^19^, ^20^, ^21^, ^22^ reported in the literature are compared with the predictions in this work and those of CSMGem.^36^ In Figure 1, the formation conditions of ethane hydrate in two hydrate systems (*I* − *H* − *V* and *L*_w_ − *H* − *V*) are shown. The quadruple point 1 (*Q*_1_) shows the temperature–pressure condition at which four phases (ice–hydrate–vapor–liquid water) are in equilibrium. The predictions in this work fit closely with all the experimental data while the predictions of CSMGem^36^ deviate from the experimental data at temperatures below −60 °C and above 15 °C.

Ethane hydrate equilibrium data in three hydrate systems (*I* − *H* − *V*, *L*_w_ − *H* − *V*, and $L_{w}-V-L_{C_{2}H_{6}}$) are shown in Figure 2. The quadruple point 2 (*Q*_2_) represent the temperature–pressure condition at which four phases (liquid water–hydrate–vapor–liquid ethane) exist together. The predictions in this work also fit closely with the experimental data at high‐pressure conditions. On the other hand, the predictions of CSMGem^36^ deviate from the experimental data at pressures above 100 MPa, as shown in Figure 2. This shows that the hydrate prediction software is not a good prediction tool for estimating ethane hydrate equilibrium data in extremely low‐temperature and high‐temperature/pressure systems. Thus, the generalized correlation developed in this work can serve as a reliable alternative for reproducing ethane hydrate equilibrium data in pure water at low, moderate, and high‐temperature/pressure systems.

The inhibiting effects of sodium chloride on the formation conditions of ethane hydrate are shown in Figure 3. Experimental data reported by Mohammadi et al.^5^ and Tohidi et al.^23^ are compared with the predictions of CSMGem^36^ and in this work. The predictions of CSMGem^36^ fit closely with the experimental data in the presence of low sodium chloride concentrations but in the presence of concentrations ≥10 wt% sodium chloride, the predictions deviate from the experimental data. On the other hand, the predictions in this work fit closely with all the available experimental data in the presence of low, moderate, and high concentrations of sodium chloride.

Ethane hydrate equilibrium conditions in potassium chloride aqueous solutions are shown in Figure 4. Experimental data reported by Mohammadi et al.^5^ and Englezos and Bishnoi^3^ are compared with the predictions of CSMGem^36^ and in this work. There is good agreement between the prediction in this work and the available experimental data. The predictions of CSMGem^36^ also fit closely with the experimental data in low concentrations of potassium chloride but deviate from the experimental data in the presence of ≥12.295 wt% potassium chloride. The prediction error of CSMGem^36^ increases with increase in the concentration of potassium chloride.

The equilibrium conditions of ethane hydrate in calcium chloride aqueous solutions are shown in Figure 5. Experimental data reported by Mohammadi et al.^5^ and Englezos and Bishnoi^3^ are compared with the predictions of CSMGem^36^ and in this work. The predictions in this work are also very close to the available experimental data and are better than those of CSMGem^36^ especially in the presence of high concentrations of calcium chloride. The prediction error of CSMGem also increases with increase in calcium chloride concentrations. This shows that the correlation developed in this work is a better prediction tool compared with other hydrate prediction software. The generalized correlation is also simple, easy‐to‐use, and does not involve rigorous computations unlike the statistical thermodynamic models available in the literature.

The formation conditions of ethane hydrate in magnesium chloride aqueous solutions are shown in Figure 6. The predictions in this work are compared with experimental data reported by Long et al.^6^ The predictions of CSMGem^36^ are not included because it cannot be used to predict the equilibrium conditions of hydrate in magnesium chloride aqueous solutions. The predictions of the generalized correlation are in satisfactory agreement with the available experimental data. Thus, it can be inferred that the generalized correlation is an excellent prediction tool for estimating the equilibrium conditions of ethane hydrate in pure water and aqueous salts solutions at extremely low‐temperature and high‐temperature/pressure conditions.

Propane hydrate equilibrium data in pure water system for two hydrate systems (*I* − *H* − *V* and *L*_w_ − *H* − *V*) are shown in Figure 7. Experimental data^4^, ^17^, ^24^, ^25^, ^26^, ^27^, ^28^, ^29^, ^30^, ^31^, ^32^ reported in the literature are compared with the predictions in this work and those of CSMGem.^36^ The quadruple point 3 (*Q*_3_) signifies the temperature–pressure condition at which four phases (ice–hydrate–vapor–liquid water) are in equilibrium. The predictions in this work fit closely with all the available experimental data while the predictions of CSMGem^36^ deviate from the experimental data at temperatures below 0 °C, as shown in Figure 7. This shows that the hydrate prediction software is not a good prediction tool for estimating propane hydrate equilibrium conditions at temperatures below the ice point.

The inhibiting effects of sodium chloride on propane hydrate formation conditions are shown in Figure 8. Experimental data reported by Patil^30^ and Tohidi et al.^23^ are compared with the predictions of CSMGem^36^ and in this work. The predictions of CSMGem^36^ deviate from the experimental data in the presence of concentrations ≥10 wt% sodium chloride and its prediction error increases with increase in the concentration of sodium chloride. On the other hand, the predictions in this work fit closely with all the available experimental data in the presence of low, moderate, and high concentrations of sodium chloride.

The equilibrium data of propane hydrate in potassium chloride aqueous solutions are shown in Figure 9. Experimental data reported by Mohammadi et al.^5^ and Tohidi et al.^23^ are compared with the predictions of CSMGem^36^ and in this work. The predictions in this work also fit closely with all the available experimental data and are better than those of the commercial hydrate prediction software. The prediction error of this software also increases with increase in the concentration of potassium chloride. This software should not be used to predict the equilibrium pressure of propane hydrate in the presence of ≥10 wt% potassium chloride solutions. The generalized correlation developed in this work can be used to reproduce propane hydrate equilibrium conditions in low, moderate, and high concentrations of potassium chloride solutions.

Propane hydrate equilibrium conditions in calcium chloride aqueous solutions are shown in Figure 10. Experimental data reported by Mohammadi et al.^5^ and Tohidi et al.^23^ are compared with the predictions of CSMGem^36^ and in this work. The predictions in this work are also very close to the available experimental data and are better than those of CSMGem^36^ especially in the presence of high concentrations of calcium chloride. The prediction error of CSMGem also increases with increase in calcium chloride concentrations. It can be inferred that the simple correlation developed in this work is also a good prediction tool for estimating the equilibrium data of propane hydrate in pure water and aqueous solutions of sodium chloride, potassium chloride, and calcium chloride at low, moderate, and high concentration, temperature, and pressure conditions.

Isobutane hydrate equilibrium conditions in pure water system for two hydrate systems (*I* − *H* − *V* and *L*_w_ − *H* − *V*) are shown in Figure 11. Experimental data^1^, ^29^, ^32^, ^33^, ^34^ reported in the literature are compared with the predictions in this work and those of CSMGem.^36^ The quadruple point 4 (*Q*_4_) represents the temperature–pressure condition at which four phases (ice–hydrate–vapor–liquid water) exist together. The predictions of CSMGem^36^ deviate from the experimental data at temperatures below the freezing point of water while the predictions in the work fit closely with all the available experimental data. This shows that the hydrate prediction software is not a good prediction tool for estimating isobutane hydrate equilibrium data in pure water at temperatures below the ice point.

The equilibrium conditions of isobutane hydrate in sodium chloride aqueous solutions are shown in Figure 12. Experimental data reported by Schneider and Farrar,^33^ and Rouher^35^ are compared with the predictions of CSMGem^36^ and in this work. The predictions in this work are also very close to the available experimental data and are better than those of CSMGem.^36^ The prediction of CSMGem deviate from all the experimental data and its prediction error increases with increase in sodium chloride concentrations. It can be summarized that the correlation developed in this work is also a good prediction tool for estimating the equilibrium data of isobutane hydrate in pure water and sodium chloride aqueous solutions.

The predictions in this work fit closely with all the experimental data reported in the literature and are better than the predictions of CSMGem.^36^ The average deviation of the predicted hydrate equilibrium pressures (AADP%) of CSMGem and the correlation developed in this work are shown in **Table** **6**. The overall AADP (%) of the generalized correlation developed in this work and CSMGem^36^ are 1.36 and 7.30, respectively. This shows that the generalized correlation is an excellent prediction tool for estimating the equilibrium conditions of ethane, propane, and isobutane hydrates in pure water and aqueous salt solutions at extremely low‐temperature and high‐temperature/pressure conditions.

**Table 6:** Average deviations of the predicted hydrate equilibrium pressures (AADP%) in different solutions

Hydrate former	Solution	*T* range [°C]	*P* range [MPa]	Data points	Reference	AADP (%)	
						CSMGem	This work
Ethane	Pure water	−28.25 to −1.25	0.122 to 0.443	10	Yasuda and Ohmura^4^	1.51	2.42
		0.25 to 13.85	0.545 to 3.054	11	Roberts et al.^15^	5.08	2.45
		17.27 to 25.21	19.48 to 83.75	24	Nakano et al.^21^	7.03	2.31
		24.86 to 50.78	89.0 to 479.0	20	Morita et al.^22^	6.95	1.25
	10 wt% NaCl	0.55 to 6.90	0.883 to 2.165	5	Tohidi et al.^23^	6.27	0.07
	10 wt% KCl	−2.75 to 8.45	0.50 to 2.11	6	Mohammadi et al.^5^	2.50	0.73
	15 wt% CaCl_2_	−5.98 to 2.05	0.573 to 1.613	5	Englezos and Bishnoi^3^	15.44	0.39
	7.62 wt% MgCl_2_	6.15 to 10.15	1.52 to 2.70	5	Long et al.^6^	‐	0.37
Propane	Pure water	−25.25 to −11.05	0.048 to 0.099	8	Holder and Godbole^32^	12.4	1.36
		−11.95 to −0.25	0.100 to 0.172	7	Deaton and Frost^25^	11.53	1.20
		0.05 to 4.85	0.165 to 0.472	10	Miller and Strong^24^	1.92	1.20
		1.05 to 5.25	0.207 to 0.542	9	Kubota et al.^28^	4.95	3.51
	3 wt% NaCl	−0.95 to 3.05	0.179 to 0.455	4	Patil^30^	4.37	2.41
	5 wt% KCl	−1.15 to 3.05	0.18 to 0.46	4	Mohammadi et al.^5^	2.41	0.08
	15.2 wt% CaCl_2_	−6.75 to −5.15	0.234 to 0.359	5	Tohidi et al.^23^	23.12	1.12
I‐butane	Pure water	−38.39 to −0.02	0.009 to 0.120	34	Buleiko et al.^1^	8.36	1.97
		0.05 to 1.85	0.115 to 0.169	15	Rouher and Barduhn^34^	4.11	0.90
		0.05 to 1.95	0.11 to 0.167	9	Schneider and Farrar^33^	2.18	1.82
	1.1 wt% NaCl	0.05 to 1.05	0.127 to 0.160	6	Schneider and Farrar^33^	5.17	0.47
	5 wt% NaCl	−3.15 to −1.50	0.105 to 0.142	8	Rouher and Barduhn^34^	13.41	1.22
Overall				205		7.30	1.36

The absolute average deviations of the hydrate equilibrium pressure (AADP)% were determined by using Equation (10). In the equation, *N*_op_ is the number of data points, *P*_cal_ (MPa) is the equilibrium pressure calculated using either CSMGem, Multiflash, or the developed correlation, and *P*_exp_ (MPa) is the equilibrium pressure determined experimentally as reported in the literature

(10) $$\mathrm{AADP}\left(\%\right)=\frac{1}{N_{\mathrm{op}}}\sum_{i=1}^{N_{\mathrm{op}}}\left[{\left|\frac{P_{\mathrm{cal}}-P_{\exp}}{P_{\exp}}\right|}_{i}\right]\times 100$$

## 4 Conclusion

A generalized correlation was developed for predicting the equilibrium conditions of ethane, propane, and isobutane hydrates in pure water and aqueous solutions of sodium chloride, potassium chloride, calcium chloride, and magnesium chloride. The generalized correlation is applicable to extremely low temperature and moderate and high temperature/pressure conditions. The predictions of the generalized correlation are in excellent agreement with all the available experimental data in the literature. The predictions in this work are more accurate and better than the predictions of the commercial hydrate prediction software. The generalized correlation is strongly recommended for the prediction of hydrate equilibrium data in pure water and aqueous salt solutions at low and high‐temperature/pressure conditions, especially in the deepwater/ultra‐deepwater areas. It can also be used to calculate the specific amount of salt required to prevent hydrate formation while drilling through oil and gas formations or hydrate‐bearing sediments.

## Conflict of Interest

The authors declare no conflict of interest.
